# Supplementary material for: Effect of orally administered cannabidiol oil on daily tonometric curve in healthy Italian Saddle horses
Source: PLoS One. 2025 May 28;20(5):e0325191. doi: 10.1371/journal.pone.0325191 (PMC12118931; doi:10.1371/journal.pone.0325191)
Supplement: Attachment — Submitted filename: comments for authors.docx [file pone.0325191.s002.docx]

The manuscript entitled “Effect of orally administered cannabidiol oil on daily tonometric curve in healthy Italian Saddle horses.” investigate the use of a natural substance such as cannabis oil to evaluate the effects of such administration on eye pressure trends in the horse. This work is very interesting, in terms of evaluating the health status of the horse's eye but also as it relates to the diagnostic aspect of eye diseases. It is well structured but some parts turn out to be written in too superficial and shallow a manner to be a scientific work.

In my opinion this work should be deepened and revised in many parts, and rewritten in the discussion, also following the advice and comments below following a major revision.

**Introduction**

Line 49 Please unify the number of bibliographical sources and dots through the main text

Line 45-54 As the title of the present article suggest that you were talking about healthy subjects, first of all I suggest to describe the physiological oscillation of the IOP for example after exercise in horses, for example include this source to deep the section:

doi: 10.3390/ani12141850.

Line 49-55 this section is described too broadly and in too little depth, so the arguments regarding therapeutic approaches are disconnected with the introductory section

Line 56-57 some new bibliographic source to accompany the section should be included as:

<https://doi.org/10.3389/fvets.2024.1496473>

<https://doi.org/10.3389/fvets.2024.1341396>

**Materials and methods**

Line 91-92 if the horses were already stabled in the mentioned way, according to precise routines, what was the purpose of the 5-day acclimatization period? Please be more specific and specify what the horses needed to acclimatize to

Line 92-93 With what instrumentation the IOP was measured and by what methodology

Line 101-102 The product manufacturer should be reported

**Results and Discussion**

Line 116-117 data were normalized results or not? this result should be reported

Line 135-136 The mention both eyes and respectively right and left is redundant

Line 137 the same mention “No effect of sex was found” is redundant compared to lines 133-134

Line 116-121 In addition, a two-way ANOVA was performed in the statistical analysis. Better clarify which variables were evaluated. Also, in the results section these significant values should be detailed. To establish a significant peak over time, a post hoc analysis should also have been performed, so describe it accurately both in the statistical analysis section and in the results.

Results in too little content. According to when IOP measurements over time were assessed before CBD administration and then 3 times every 8 days. Why does it appear in Figure 1 only before and after administration. Please clarify these concepts so that they are easy for a reader to interpret.

Table 1 If results were calculated and expressed as mean and standard deviation, this information should be specified in the results rection

Line 143-144 Avoid repetitions

Line 151 Please mention the examples reported in horses as showed in the previously suggested bibliographical sources

Line 155 nothing was previously mentioned about THC use or information in the text. Please explain its importance and usage even in other sections or delete it.

The importance of eye pressure changes in both physiological and pathological settings should be emphasized.

The discussions are too short and poor in content. there is described only what has been found in the literature on the topic. The discussion section should be a discussion regarding the results obtained, so one should first describe what was obtained, comment on its possible meanings, and discuss comparison with other studies that found similar or opposite results based on the variables analyzed, so the section is all in need of rewriting

Conclusions should also more emphasize the results obtained and indicate in what ways these results can be useful in the scientific community
