# Supplementary material for: Effect of orally administered cannabidiol oil on daily tonometric curve in healthy Italian Saddle horses
Source: PLoS One. 2025 May 28;20(5):e0325191. doi: 10.1371/journal.pone.0325191 (PMC12118931; doi:10.1371/journal.pone.0325191)
Supplement: Attachment — Submitted filename: Response to Reviewers.docx [file pone.0325191.s005.docx]

**Dear Editor and Reviewers, on behalf of all other Authors, I would thank for the time and efforts spent in reviewing the manuscript and for the points raised, which allow to improve the overall quality of the paper. We have carefully considered your suggestions and addressed them throughout thew text, using the bold blue type to highlight changes in the text.**

**In addition, English language has been reviewed by a native speaker.**

**Below, in bold blue type, the point-to-point reply to reviewers**

Reviewer #1: Please see the attached file.
The manuscript entitled “Effect of orally administered cannabidiol oil on daily tonometric curve in healthy Italian Saddle horses.” investigate the use of a natural substance such as cannabis oil to evaluate the effects of such administration on eye pressure trends in the horse. This work is very interesting, in terms of evaluating the health status of the horse's eye but also as it relates to the diagnostic aspect of eye diseases. It is well structured but some parts turn out to be written in too superficial and shallow a manner to be a scientific work.
In my opinion this work should be deepened and revised in many parts, and rewritten in the discussion, also following the advice and comments below following a major revision.

The manuscript entitled “Effect of orally administered cannabidiol oil on daily tonometric curve in healthy Italian Saddle horses.” investigate the use of a natural substance such as cannabis oil to evaluate the effects of such administration on eye pressure trends in the horse. This work is very interesting, in terms of evaluating the health status of the horse's eye but also as it relates to the diagnostic aspect of eye diseases. It is well structured but some parts turn out to be written in too superficial and shallow a manner to be a scientific work.

In my opinion this work should be deepened and revised in many parts, and rewritten in the discussion, also following the advice and comments below following a major revision.

**Introduction**

Line 49 Please unify the number of bibliographical sources and dots through the main text **Text has been modified to reflect this comment**

Line 45-54 As the title of the present article suggest that you were talking about healthy subjects, first of all I suggest to describe the physiological oscillation of the IOP for example after exercise in horses, for example include this source to deep the section:

doi: 10.3390/ani12141850. **Text has been modified to reflect this comment and reference has been added**

Line 49-55 this section is described too broadly and in too little depth, so the arguments regarding therapeutic approaches are disconnected with the introductory section **Section rephrased to ensure more clarity**

Line 56-57 some new bibliographic source to accompany the section should be included as:

<https://doi.org/10.3389/fvets.2024.1496473>

<https://doi.org/10.3389/fvets.2024.1341396>

**Text has been modified to reflect this comment and references have been added**

**Materials and methods**

Line 91-92 if the horses were already stabled in the mentioned way, according to precise routines, what was the purpose of the 5-day acclimatization period? Please be more specific and specify what the horses needed to acclimatize to **Lines implemented in the manuscript and a proper reference has been added**

Line 92-93 With what instrumentation the IOP was measured and by what methodology **IOP was evaluated with Tonopen Vet, Reichert Inc., Depew, NY USA. Detailed in the text**

Line 101-102 The product manufacturer should be reported

**Results and Discussion**

Line 116-117 data were normalized results or not? this result should be reported

Line 135-136 The mention both eyes and respectively right and left is redundant

Line 137 the same mention “No effect of sex was found” is redundant compared to lines 133-134

Line 116-121 In addition, a two-way ANOVA was performed in the statistical analysis. Better clarify which variables were evaluated. Also, in the results section these significant values should be detailed. To establish a significant peak over time, a post hoc analysis should also have been performed, so describe it accurately both in the statistical analysis section and in the results.

Results in too little content. According to when IOP measurements over time were assessed before CBD administration and then 3 times every 8 days. Why does it appear in Figure 1 only before and after administration. Please clarify these concepts so that they are easy for a reader to interpret.

Table 1 If results were calculated and expressed as mean and standard deviation, this information should be specified in the results rection

Line 143-144 Avoid repetitions

Line 151 Please mention the examples reported in horses as showed in the previously suggested bibliographical sources

Line 155 nothing was previously mentioned about THC use or information in the text. Please explain its importance and usage even in other sections or delete it.

The importance of eye pressure changes in both physiological and pathological settings should be emphasized.

The discussions are too short and poor in content. there is described only what has been found in the literature on the topic. The discussion section should be a discussion regarding the results obtained, so one should first describe what was obtained, comment on its possible meanings, and discuss comparison with other studies that found similar or opposite results based on the variables analyzed, so the section is all in need of rewriting

Conclusions should also more emphasize the results obtained and indicate in what ways these results can be useful in the scientific community

**Statistical analysis has been run again, applying both a mixed effect model analysis and post hoc analysis to reflect your recommendations, so the paragraph has been rewritten accordingly. Results, Discussion and Conclusion sections have been completely rewritten to be more specific and consistent, incorporating all your suggestion**

Reviewer #2: I have read and reviewed the manuscript entitled: Effect of orally administered cannabidiol oil on daily tonometric curve in healthy Italian Saddle horses. Overall, from this reviewer’s perspective, this is an interesting study; however, in its current state, it shows many deficiencies. For example, the methodology section requires an orderly and detailed description to ensure that the study can be replicated. Regarding the statistical analysis, I suggest the authors modify it to a mixed linear model that allows the establishment of statistically significant differences between treatments and the various measurement moments, in addition to correctly analyzing the tonometric curve on the three occasions in which the measurements were repeated. On the other hand, the lack of a control group generates an important bias in the research. Finally, I would like to highlight that the discussion section should be rewritten. This is to explain the neurophysiological events that occur under the effect of CBD to reduce intraocular pressure.Likewise, other observations must be addressed to achieve publication quality. I have left some comments, hoping that they can help the authors.

**Thanks for your insights and comments. We have amended the text in the proper parts to reflect your suggestions.**


General comments
L31-33: Please indicate the IOP values before and after CBD administration, with their respective P values. **Text modified to reflect this comment**


L66: CBD has also been used in pets for the control of acute pain caused by an ovariohysterectomy and chronic pain caused by osteoarthritis. Please add the following references to your manuscript, which you can also use in the discussion of your manuscript:
10.3389/fvets.2024.1380022
10.3389/fvets.2022.1050884

**Text modified to reflect this comment**

L69: I suggest that the authors add the following references in this line:
10.3389/fvets.2024.1496473
10.3389/fvets.2024.1341396
10.1016/j.jevs.2019.102880

**Text modified to reflect this comment**

L73: Please add a hypothesis. **Text modified to reflect this comment**

L82: What was the statistical method used to determine the sample size? Please clarify.

**The sample size needed was calculated with Sealed Envelope™ calculator, setting significance level at 5% and power at 95%, detailed in the text**

L84: Please indicate which exclusion criteria were considered in your study. **Text modified to reflect this comment**

L85: What were the analytes or parameters measured in blood before the start of the study? And how long before the administration of CBD were these blood studies performed? **Text modified to reflect this comment**

L108: Due to the type of experimental design used, I suggest the authors perform a statistical analysis based on a linear mixed model instead of a 2-way ANOVA. This model will allow the analysis of the differences observed between treatments and evaluation times, and even for the analysis of the tonometric curve (comparison of three measurements) with their respective washout periods. **Statistical analysis has been run according to your suggestion and** **text modified to reflect this comment**

L123: Table 1, please indicate the statistical differences observed between treatments and evaluation times with letters and numbers. In this sense, also the results obtained after the administration of CBD at 15 minutes, 30 minutes, 60 minutes, 2 hours, 3 hours, 4 hours, 6 hours, and 8 hours should be integrated into this table; although Figure 1 already shows this information. **Table 1 has been updated to reflect your comment**

L141: In general, the discussion is a section that is shown to be deficient. Therefore, I suggest that the authors complement this section. For example, the neurophysiological effects that generate the decrease in intraocular pressure under the effect of CBD should be explained. I suggest consulting and citing the following references:
10.2460/javma.24.06.0360
10.3934/Neuroscience.2024009
10.3390/ph17060748
10.1016/j.ijpharm.2022.121627
10.3390/ijms22073798
10.1016/j.survophthal.2020.07.002
10.1097/01.ijg.0000212260.04488.60
10.3389/fvets.2022.1050884
10.3390/ph16081149
10.1016/j.biopha.2022.112981
10.1055/a-1665-3100
10.1016/j.exer.2020.108266
10.2174/1570159X15666170724104305
10.1016/j.biopha.2016.11.106
10.1155/2016/9364091
10.1016/S0079-6123(08)01131-X
<https://pubmed.ncbi.nlm.nih.gov/34283478/>

L167: I suggest that the authors discuss the limitations and perspectives of their study.

L171: Conclusions must be rewritten based on changes arising from modifications made to the statistical analysis.

**Discussion and Conclusion sections have been completely rewritten to be more specific and consistent, incorporating all your suggestion**
