# Supplementary material for: Effect of orally administered cannabidiol oil on daily tonometric curve in healthy Italian Saddle horses
Source: PLoS One. 2025 May 28;20(5):e0325191. doi: 10.1371/journal.pone.0325191 (PMC12118931; doi:10.1371/journal.pone.0325191)
Supplement: Attachment — Submitted filename: Reviewer 3.docx [file pone.0325191.s004.docx]

I reviewed the edited version of the manuscript and provide the following comments to the authors. The majority of my comments are intended to correct the manuscript for grammatical errors and clarity.

Line 52/53 – suggest changing to “…common and represents a major cause of retinal….
Line 65 – suggest rewording the section “determine a lowering in IOP” to be more clear and understandable.
Line 74 – 76 – suggested edit “In the last few years…investigated in pet medication, …”
Line 116 – how was the length of the washout period determined? This information should be added to the manuscript.
Line 165 – suggested edit “….after one hour post CBD administration…left eyes) and maintaining….”
Line 178 – 180 – this sentence should be reworded to expand and provide clarity on the intended information this sentence is adding to the manuscript.
Line 188 – suggested edit “…was started to be studied for its…”
Line 190 – edit to “…the insight into the mechanism….”
Line 201 – 207 – this section needs to be reworded to ensure clarity for the reader.
Line 231 – 233 – please expand this section so that the point you are making is clear to the reader.
